# Biomaterial Enhanced Regeneration Design Research for Skin and Load Bearing Applications

**DOI:** 10.3390/jfb10010010

**Published:** 2019-01-26

**Authors:** Dale S. Feldman

**Affiliations:** UAB, Department of Biomedical Engineering, University of Alabama at Birmingham, Birmingham 35294, AL, USA; dfeldman@uab.edu

**Keywords:** Biomaterial enhanced regeneration, skin regeneration, fracture fixation, degradable/regenerative scaffolds

## Abstract

Biomaterial enhanced regeneration (BER) falls mostly under the broad heading of Tissue Engineering: the use of materials (synthetic and natural) usually in conjunction with cells (both native and genetically modified as well as stem cells) and/or biological response modifiers (growth factors and cytokines as well as other stimuli, which alter cellular activity). Although the emphasis is on the biomaterial as a scaffold it is also the use of additive bioactivity to enhance the healing and regenerative properties of the scaffold. Enhancing regeneration is both moving more toward regeneration but also speeding up the process. The review covers principles of design for BER as well as strategies to select the best designs. This is first general design principles, followed by types of design options, and then specific strategies for applications in skin and load bearing applications. The last section, surveys current clinical practice (for skin and load bearing applications) including limitations of these approaches. This is followed by future directions with an attempt to prioritize strategies. Although the review is geared toward design optimization, prioritization also includes the commercializability of the devices. This means a device must meet both the clinical performance design constraints as well as the commercializability design constraints.

## 1. Introduction

This review is meant to help tie together the research papers in this special edition issue. The goal is to define the field of biomaterial enhanced regeneration (BER) and provide examples of the types of research in the field, in selected areas. The examples are to help justify the inclusion of the papers in this issue, not to suggest that they are the best approaches. The examples will come predominantly from my research and will include research found in most of the papers in this issue. Although the research approaches and design optimization studies will be justified, the justification will be why they are reasonable approaches not that they are necessarily the best approaches nor the only way to meet the clinical performance constraints.

### 1.1. Scope of Field

Biomaterial enhanced regeneration (BER) falls mostly under the broad heading of Tissue Engineering: the use of materials (synthetic and natural) usually in conjunction with cells (both native and genetically modified as well as stem cells) and/or biological response modifiers (growth factors and cytokines as well as other stimuli, which alter cellular activity). The goal is to use these systems to replace tissue and organ functions (biochemical and/or structural).

Biomaterial enhanced regeneration is the branch of Tissue Engineering that puts the emphasis on the functional biomaterial. This is the designing of solid materials to help in the regenerative process. The biomaterial could deliver and/or protect added cells (also potentially guiding the differentiation of stem cells) or biological response modifiers, serve as a scaffold, or help activate cells by other mechanisms all in an effort to help promote the healing and regenerative process.

Enhancing regeneration is not only moving more toward regeneration but also speeding up the process. Typically, the rate limiting step for regeneration is angiogenesis (ingrowth of blood supply into a biomaterial) to provide short and long-term viability of the tissue as well as a high enough oxygen level for fibroblasts to produce extracellular matrix.

One way to have the functional biomaterial enhance the regenerative process is to serve as a tissue scaffold. The presence of a scaffold reduces the time of healing by reducing the need for the fibroblasts to produce the scaffold along which it can migrate. The scaffold can also help to better utilize biological response modifiers that stimulate mitosis or migration, to provide a surface for cells to attach to and move along.

### 1.2. Scope of the Review

The goal is to be comprehensive on design principles and general strategies for regenerative biomaterials as well as options for design optimization. This is in an effort to further define the field of biomaterial enhanced regeneration (BER) as well as research areas for design optimization. Since different applications have different clinical needs and therefore potentially different specific design strategies, the review will focus on only two applications: skin defects and load bearing biomaterials. The review will also just concentrate on regenerative systems. The goal for this section is also helping to define the field and types of research that can be done. Again, the research approaches and design optimization studies are to provide examples of research in BER, not to suggest these are the best or only approaches to meet the clinical performance constraints.

For skin, the review will concentrate on full-thickness skin defects with pressure ulcers in the spinal cord injured and burns the two main applications. For load bearing applications, the review will concentrate on fractures in the central portion of long bones that require internal fixation. The review will also briefly cover current clinical strategies (at the beginning of the 21st century) and their limitations as well as potential future directions.

### 1.3. Principles

#### 1.3.1. Design Hierarchy

In order to use functional biomaterials to their fullest, it is critical to determine the optimal implant design strategy for each application. A biocompatibility hierarchy can be developed and used to help determine the optimal implant design strategy in each case [1,2,3,4]. The optimal theoretical design strategy would be at the top of the hierarchy, however limits on current technology and the commercializability of the design can change what would be an optimal practical design as well as an optimal marketable design [4]. Biomaterial enhanced regeneration design research would typically be how to move up the design hierarchy by developing new technology (or using technology not used currently in clinical practice) to meet or exceed the clinical performance requirements. The clinical performance can be a better clinical outcome (e.g., healing, recovery of function, esthetics, etc.) or strategies to make the functional biomaterial commercializable [4].

To develop a biocompatibility hierarchy it is important to understand what biocompatibility is and how it is determined. Biocompatibility is the study of how the host affects the implant (e.g., corrosion and degradation) and how the implant affects the host (e.g., inflammation and allergic response) [4,5]. It also is used to make relative comparisons, i.e., a device is or is not biocompatible as well as this design is more biocompatible than that design [1,5]. By developing a ranking system of host and implant responses, the most biocompatible implant system can be determined and then designed for each application [1,4].

When implants were first developed, a biocompatible material was defined as one that was capable of being implanted; caused no systemic toxic reaction; having no carcinogenic qualities, and the local tissue response of which neither compromised function nor caused pain, swelling, or necrosis [2]. This definition tells us what an inert biomaterial cannot do, not what a functional biomaterial should do.

Since then, biocompatibility has been viewed in many different ways. In the 1970s biocompatibility was described as an interfacial problem [1]. The body interacts with the surface of the biomaterial, which can differ from the bulk of the material. This emphasis led to the critical attention to surface analysis and characterization that is still a major part of biomaterials research today.

More recently, the definition has changed to be a more positive one:

“The ability of a material to perform with an appropriate host response in a specific application” [3].

Two difficulties with this definition, however, are:

1. Biocompatibility is dependent on time. An implant can be biocompatible for short-term applications, but not long-term ones. In addition, an implant may trigger a “bad” (or inappropriate) response in the short-term in order to elicit a “good” (appropriate) response in the long-term [4].
2. An “appropriate response” is much better than an “inert response”, but still does not provide much guidance on what is “appropriate”.

This can be solved by a more design driven one: as what one wants the response to be in a particular situation [4,5]. For example:

“The ability of a biomaterial to perform its desired function with respect to medical therapy… generating the most appropriate beneficial cellular or tissue response in that specific situation, and optimizing the clinically relevant performance of that therapy” [6].

The first part is a good definition, but the middle part implies perfection or the best response [4]. In many cases, however, current technology does not allow perfection without some “inappropriate responses” [4]. Also, although the goal should be optimization as in the last part, improvement does not make something biocompatible. The best definition, I believe, is the first part—to perform as designed. Does it meet the design constraints we set out (both positive (clinical performance requirement) and negative (limit on inappropriate responses)) [4]. This does however put the onus on us to decide what the device should do, as a minimum [4]. The problem is in developing a device we have to look at *in vitro* and *in vivo* bioprocesses and decide the design constraints for the bioprocesses to meet the clinical performance [7].

Therefore, our constraints for “as designed” will change as we determine, if our *in vitro* design constraints meet our *in vivo* design constraints and if our *in vivo* design constraints lead to the desired clinical performance [7]. In general, however, we can look at a design hierarchy for the material and host response (Table 1).

Table 1 shows some general design strategies for functional biomaterials used for biomaterial enhanced regeneration. It is broken into host response and implant response. Under each category potential responses are listed with “inert” at the bottom of the hierarchy. The implant can be inert or be modified in the biological environment as well as the host response can be inert or the biomaterial can induce a tissue response. Any of these levels can be deemed biocompatible, if it is the desired host and implant response believed to achieve the desired clinical response [4]. There are of course many bioprocesses to optimize for each level and some designs span multiple levels [7]. In general, however, the top of the hierarchy (degradable/regenerative systems) would provide the best clinical response [8]. There are many reasons the top of the hierarchy is not the clinical gold standard for an application, e.g.; the difficulty to commercialize it, our inability to achieve regeneration, the need for long-term mechanical stability; or the ability to meet the clinical design constraints at a lower level on the hierarchy [7,8].

Inert implants are the most common type of implants currently on the market [7,8]. Most implants like artificial joints are designed to serve a function without being altered in any way [8]. The implant, however, can be designed to be modified *in vivo*. For example, it can be surface active having a bioglass, calcium phosphate, or biochemically active surface that can stimulate an *in vivo* response [1,8]. One example of this surface activity is the use of hydroxyapatite or bioglass coatings on orthopedic and dental implants to get better bonding to bone [1]. The implant can also serve as a drug delivery system for biochemical agents as in wound dressings that release antibiotics or growth factors [4,8]. Alternatively, the implant can serve its function and dissolve away, like degradable sutures [4].

Similarly, the implant can stimulate an inert host response or an active response. Again, most implants presently on the market are designed to be inert in the host environment (the original definition) [4,7,8]. They perform a function with as little modification of the host as possible. Few are truly inert and the goal is to limit the inflammatory response at least in the long-term [7].

In many cases, however, it is beneficial to have the implant integrated with the host [7]. A porous implant can be used to stimulate tissue ingrowth [4]. A bioactive calcium phosphate surface has been used to get direct bone attachment [1]. Both of these responses have been used to achieve better long-term stability [7]. Finally, some implants are designed to trigger a regenerative response. Since bone is regenerative, fracture-fixation systems such as bone plates are designed to heal fractures by regeneration [7].

Although the field of Biomaterials has come a long way, and many responses can be stimulated, we are still not capable of completely duplicating the structure and function of the replaced part [4,7]. Note in this review, this will be emphasized in our inability to produce skin graft substitutes; but are better served to make scaffold systems [4,7]. In addition, man-made materials will lose part of their function and properties over time and cannot "heal" like biological tissue [7]. Therefore, the best biocompatible response is for the implant to stimulate tissue regeneration [4]. For regeneration to work, however, the implant must resorb or degrade as it stimulates regeneration—the process of biomaterial enhanced regeneration [4,8].

In addition, the best design is where the degradation and regeneration occur at the same rate [7]. This has been called isomorphous tissue replacement [9]. If degradation proceeds too quickly, the biomaterial loses its ability to serve as a tissue scaffold and the biologic tissue scaffold regenerated is not complete before the biomaterial is gone [7]. If the regeneration rate is faster than the degradation rate, the matrix will slow the regenerative process and become the rate-limiting step [7]. To achieve isomorphous tissue replacement for synthetic degradable materials as well as artificially crosslinked natural materials the degradation rate must be adjusted to match the healing rate [10]. The ultimate design, however, would be a system where the regenerative process controls the degradation [7]. This would allow the system to automatically take in to account patient-to-patient variability in healing rate, adjust healing rate continually rather than just approximate the overall rate, and could be easily modified for use in different tissues with different healing rates [7]. This biofeedback control can occur in a number of ways. One example would be how the body normally does this: as the cells come in to repair the tissue they breakdown the provisional matrix [7,11].

The hierarchy in Table 1 has the degradable/regenerative response at the top (with them being tied together) as a first choice design [7]. Sometimes, however, the tissue cannot regenerate adequately with current technology or Food and Drug Administration (FDA) approved materials, so different options down the hierarchy should be selected first. The goal of a degradable/regenerative system can be applied in virtually every application, although some systems are further along than others are. Although each application may start at a different place on the hierarchy, they all should continually move upwards.

#### 1.3.2. Emphasis

The emphasis for this review will be on the top of the hierarchy—biomaterials that degrade and help the regenerative process. Although most of the topics covered are applicable to all types of implants, the target applications are skin and load bearing devices; with a state of the art section at the end of the review. Again, the intent is to be comprehensive on general strategies for designing, but just citing a limited number of specific examples. Also the emphasis will be on challenges for the general strategies as opposed to comparing the effectiveness of specific techniques or approaches. In some cases, study results will be presented, but mostly related to general strategies versus comparisons between specific designs.

One of the big challenges in Tissue Engineering is the tradeoff between effectiveness and development cost (including obtaining regulatory approval). Simplistically the more biological things added to the biomaterial (e.g., biochemical (including growth factors) and cells) the longer and more costly the development process. This review, however, will concentrate more on effectiveness of designs and strategies versus ease of commercialization, although cost-effectiveness will be part of the justification for some strategies.

The hierarchy presented is from an effectiveness perspective. From a design standpoint, the commercialization issues can be put as design constraints and alter the desirability of one level of the hierarchy over another. For some applications complete regeneration of structure or function is not necessary, although it might be the most effective solution. In many cases, once the design constraint is reached, the benefit of improving the efficacy is not worth the additional development costs [7]. The tradeoffs between effectiveness and commercializability can change from application to application as well as from company to company. Also over time the development costs can be reduced by things like advances in technology or regulatory approval of similar devices to change the cost versus benefit analysis [7,8,11].

#### 1.3.3. Responsibility for Design Studies

Another reason the emphasis will be on general strategies versus specific designs is the lack of clinical data for most of these designs, and therefore the limited information on clinical efficacy. Although it is difficult to get funding for or publish articles that are in the final stages of product development, a specific research paper should be able to place the research in the continuum of steps toward the development of a marketable product [12,13].

This is important for justification; justification of the need for the study, the approach used, and the significance of the results [12]. It depends, to a degree on the type of study and where in the design process it fits. An applied paper should be design driven, even if it is written as hypothesis driven. There should therefore be design constraints, what the design should be able to do at a minimum. The study should explain where it fits in meeting these design constraints [12,13]. 

Although “evidenced-based medicine” is based on the scientific method, there are a few things necessary (that are not required for the scientific method) to prove a design is effective or better than current treatment [12,13]. In order to better understand how design constraints fit in, it would be helpful to explain them in terms of the engineering design process including the commercializability. First is in establishing a problem [13]. This is in two parts: (1) how far short of the needed clinical parameters are current treatments and (2) how significant a problem is this? In essence a cost/benefit analysis: is the potential benefit of the solution worth the cost and time to develop as well taking into account any associated side effects [12,13]. Part of this is determining how big a difference in clinical performance would actually make a difference (significant clinical impact) [13]. 

After establishing the problem, the design constraints can be developed. What would success look like? What should the design do as a minimum? Then any proposed solution needs to meet all the design constraints [12,13]. The comparison of solutions should be on the clinical significance of meeting each of these design constraints above the minimum: significantly better does not mean it is clinically better [12,13].

Although again since it is unlikely that the design process is complete, where the study fits into the process has to be justified [12]. As a minimum the problem and its significance has to be specified. Further, the specific improvement in clinical performance should be specified (as quantitatively as possible) as well as the believed relationship between all the pre-clinical performance design constraint(s) the study is focusing on [7,12,13]. It is fine, if it is a feasibility study to determine if the proposed solution has the potential to meet the pre-clinical design constraint(s), which could potentially allow it to meet the clinical performance design constraint(s); it just needs to state the purpose of the study [12]. In the discussion, what the study showed relative to the design process should be explained as well as, at least in general, what future studies are needed to determine if the proposed solution could meet the clinical performance design constraints [12,13]. Too often a research paper will claim it showed the potential of the solution to be used in a clinical situation without identifying the problem with current solutions, the improvement in clinical performance desired, or what additional studies would be needed to show the solution could meet the clinical performance design constraints [12,13].

### 1.4. Design Options

Again, the emphasis for this review will be on the top of the hierarchy—biomaterials that degrade and help the regenerative process. This section will outline types of materials and modifications that can be used to enhance the regenerative process. The next section will describe specific examples of BER approaches and research studies. This will be followed by current clinical practice for skin defects and load bearing applications.

#### 1.4.1. Materials

Degradable materials can be any of the three classes of solid materials: metals, polymers, or ceramics as well as combinations of them—e.g., composites, alloys, or mixtures [4,7,8,11,14,15,16,17,18,19,20,21]. They can be organic (carbon-based) or non-organic, synthetic or natural, biologic or from non-living sources [4]. Biological biomaterials are typically from the extracellular matrix (mostly polymers and typically hydrogels) [4,11]. Other additive biologics include cells and liquid biochemicals (mostly proteins, but also many carbohydrates, and some lipids) that are not made into solid biomaterials [4,7].

#### 1.4.2. Manufacturing Techniques

Each class of material has many manufacturing techniques, each with advantages and limitations. Concerns can be high temperatures or use of chemicals that can damage any additive components such as biologics or polymers [4,5,16,17,18]. This can also be an issue in sterilization of the material to be used as an implant. 

Obviously, an implant needs to be sterilized in some fashion before implantation. The different sterilization methods (steam, gas, etc.) can impart changes to the surface or bulk (chemical or physical) that would alter both the short and long-term host and implant response [4,7]. Therefore, the selection of sterilization method is an important part of the design process. 

The biologic materials can be separated out from the living source and used as is or be modified. This includes grafts (auto-, allo-, or xeno-grafts), which can also be used as is or processed (e.g., decellularized, demineralized, or cross-linked) [16,17,18]. In recent years 3D printing (additive manufacturing) has become more popular, since it has become more versatile and accessible [16,17,18]. Not only can all three classes of solid materials be 3D printed, but biologics are being done as well [7,16,17,21].

#### 1.4.3. Properties

There are a number of critical properties for these biomaterials, which are application dependent. These can be physical or chemical. Physical can include morphological (size and shape), mechanical (initially and over time), or stability (e.g., during storage, during sterilization, and type of degradation) properties. Chemical can relate to the surface or bulk of the material. All of these have the potential to alter the biocompatibility of the material. Skin systems have properties that are different if they are to serve as a graft versus a functional replacement [22,23,24,25,26,27,28,29]. In this review, the emphasis will be on scaffold systems that intend to get the properties at least to the functional replacement level and as close as possible to the native state. 

##### Process Modifications

Again for each class of solid material (including biologics) the process parameters can be modified to alter the critical properties. If composites and mixtures are included then the relative amounts of each component (solid or liquid (e.g., growth factors)) can be altered. For each application there would be design constraints for each of the critical properties. For most individual materials and many composites there are models that map processes to the critical properties [7]. Meaning, if it is made this way the specific physical and chemical properties would be known. For many of the degradable/regenerative scaffolds the models are still under development [7]. They typically have only been characterized *in vitro* or for animal models; so determining, if the system can meet the clinical performance design constraints still needs to be tested. 

Although there are differences in process parameters, and resultant property changes, for different systems the types of modifications are similar. For example, sometimes the biomaterial or parts of it are made in a bioreactor, made prior to surgery, or made at the time of surgery.

##### Types of Modifications

Included are some of the modifications that can be used to enhance tissue regeneration, with some of the rationale for doing so. This will include changes in structure, surface properties, mechanical properties, and handling requirements. Most of these will be included in the general design strategies section with specific examples provided in the specific strategies section. For the most part, these physical and chemical properties can be altered over a range under process control. The emphasis in this review will be to talk about the modifications versus the actual processes. Also for degradable systems the effect of any modification will normally change over time as the implant degrades. Most of the examples will be modifications done to the biomaterial with the last two (cells and drug delivery) substances that are incorporated into the biomaterial. There are other strategies that can be used to help in regeneration and are benefitted by the presence of the biomaterial, but these last two rely on the biomaterial to protect them and/or activate them. There are also cases when cells and biochemical factors are added around the biomaterial or systemically (and home to the wound), which will also be mentioned although they are not direct modifications of the biomaterial.

Changes in morphological structure can be the size, shape, or 3D arrangement [4]. Again for a degradable system the structure will change over time as the implant degrades. Typically, the application requires the implant to fit into a specific area, especially if it is to be used as a skin or bone graft substitute or scaffold. The 3D arrangement can be to mimic the normal *in vivo* composite architecture (physically and/or chemically) especially if it is to be used as graft substitute. Historically, researchers have concentrated on making the pore size, shape, and interconnectivity amenable for use as a regenerative scaffold (tissue and blood vessel ingrowth) [4,7,8]. Effort has even gone into making channels that will become the new blood vessels [7]. Again as 3D bioprinting has improved, more effort has been toward making the structures more biomimetic (morphologically and chemically) to serve more like a graft substitute versus a degradable/regenerative scaffold [4,22,23,24,25,26,27,28,29].

**Figure 1:**
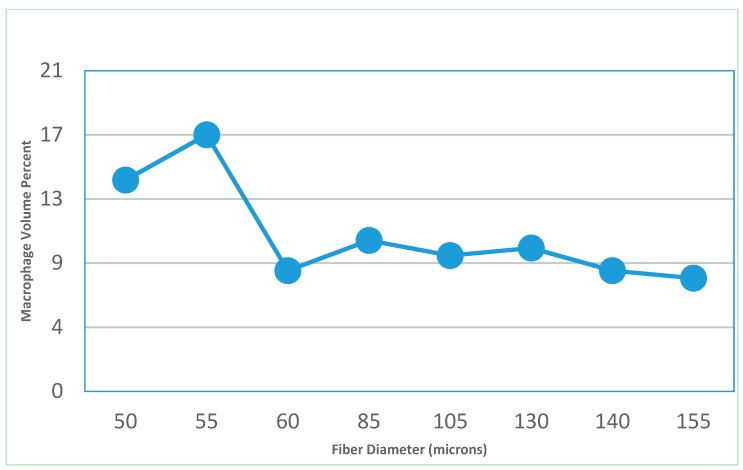
As size goes below 60 μm the macrophage response increases *in vivo*. This suggests that the inflammatory response significantly increases for diameters (polyester fibers in this case) below a certain threshold.

Size can also be important for triggering the inflammatory response [30]. If particles formed as the system degrades or parts of the scaffold have diameters below 60 μm they can increase the inflammatory response (Figure 1 [31]). This data was from an *in vivo* study using polyester fibers of different diameters. In many cases, as the size goes below a certain threshold it can decrease the response. For example, in gout as the monosodium urate crystals go below 0.5 μm they tend not to induce a macrophage response, which can trigger a gout attack [32].

Surface property changes can be chemical (including charge), shape (e.g., texturing), or density (compression of the surface). Again, for a degradable system the surface properties will change over time as the implant degrades. Chemistry changes can be used to alter the local cell attachment and activation [33]. In stable implants, without direct blood contact, proteins attach to the surface and tend to make it a non-specific response (unless there is an immune component) [7]. In blood, however, platelet activation and clotting occur quick enough that the surface chemistry is important. So a concern of a chemical surface modification is that even if it works well in cell culture, its impact clinically can be lessened by *in vivo* processes (chemical modification *in vivo* or masking of the surface via protein absorption); as well as loss of the surface as the implant degrades [7]. This degradation will also alter the surface chemistry over time. Surface changes such as coatings can be used to slow degradation until the coating is broken down [7].

Texturing can be used to allow guided cell migration or effect the thickness of the fibrous capsule formed [4]. Degradable implants can lose the advantages of surface texturing as they degrade. They also can continually create new surfaces to be exposed.

Surface density changes can be done for metals and ceramics by processes, which include ion implantation or surface compression (e.g., shot peening), which typically slow surface erosion as well as can make a material stronger. Again these surface changes only last as long as the surface layer remains intact, which tends to be a month or less for degradable metals and ceramics [7,34].

Changes in mechanical properties are usually strength and modulus (stiffness). Strength is to make sure it can handle the physiological loading, with degradable implants needing tissue ingrowth to maintain strength [7]. Stiffness has importance in a number of situations. Actually, stiffness is related to both modulus and cross-sectional area. In general, the more stiff a material is the less it deforms under the same load. There is stiffness of the surface, stiffness of the whole implant, and stiffness of the implant and tissue together. The stiffness at the surface has been shown to affect cell activity, including migration and differentiation [7,33]. Stiffness of the whole implant mostly affects the interface between the implant and surrounding tissue. Differences in deformation can cause separation or sliding of the implant relative to the surrounding tissue (which can lead to tearing). While stiffness of the whole system can determine, whether the injured tissue can get injured more or re-injured [34]. 

Although we typically think of too much loading as the cause of failure, it really is too much deformation [33]. These two are related by stiffness, with stiffer implants deforming less under the same loading. For biological tissue to get injured (partial or complete tear or break) it has deformed too much (e.g., too much separation between implant and tissue or too much deformation of a healing area) [7]. 

**Figure 2:**
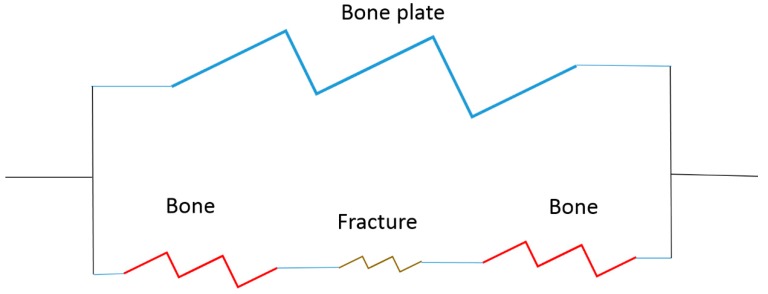
Spring model for a bone plate fixation of a fracture.

If implants and tissue are modeled as springs, too much deformation leads to a permanent lengthening (plastic deformation—tissue strain or break). The tissue or implant can be two springs in series (same load, different amounts of deformation) or in parallel (same deformation and different amounts of loading), if we use a bone plate as an example (Figure 2).

Figure 2 is a simple model of a bone plate on a fracture made of four springs. In this case the bone plate is in parallel with the bone and the bone is in series with the fracture site. Failure occurs if the fracture site deforms too much or due to stiffness mismatch there is too much sliding or separation between the bone and plate. For a degradable/regenerative system, the goal would be to maintain the mechanical properties of the system throughout the healing process with the implant stiffness decreasing over time and the fracture site stiffness increasing over time [7]. As the implant stiffness decreases, the loading on the fracture site increases. 

Handling refers to what is available at the time of surgery (including how it is prepared and stored) and how it is used at the time of surgery. For skin, which would use polymers versus ceramics or metals, implants can be made prior to surgery or made in the wound (in situ) [4]. If it is made prior to surgery, handling requirements are how to assure it fits the wound shape and how to attach it to the surrounding tissue or keep it in place. Making it in the wound (although components could be made prior to the surgery) can be made as solutions; which crosslink, polymerize, or set-up in the wound; conform to it; and adhere to it [4,7]. Handling issues would include: how to keep it in place while it is setting up and how to avoid losing biological activity in storage and during the in situ formation process (if biologics are used).

##### Stability

Although there are multiple definitions of stability, the one used here is related to resistance to changes in surface chemistry or physical properties (e.g., size and mechanical properties). Degradation is due to a lack of stability and is easier to quantify than stability. Degradation can refer to loss of material or loss of mechanical properties (the two aspects covered here) as well as changes in chemistry. The rate of degradation can be quantified for both of these and they typically occur at different rates [7]. How it degrades both determines these rates as well as the effect over time on the other modifications [7]. Typically changes in the other design options will alter the degradation as well.

The type of degradation can be classified by the way the chemical bonds are broken, whether the degradation is constant throughout or not, the size and shape of the breakdown products, and the mechanism of removal from the site [7]. Bonds can be broken by water (hydrolytic), specific chemicals (enzymes from cells is common), or other exogenous factors (e.g., heat, mechanical loading, and ultrasound) [7]. Breakdown products can be ions or other chemical compounds that go into solution or pieces of the implant that break-off. Heat or gases can also be by-products of the breakdown process. Chemicals or particles can cause local or systemic responses (toxic levels can be seen locally or where the breakdown products get sequestered). Particles typically do not cause a response unless they are between 1–60 μm [7,31,32].

Breakdown products can be cleared by the lymphatic or vascular system (some particles, however, can be trapped locally—e.g., asbestos in the lungs) [7]. The breakdown products can get to these systems via diffusion or cells (typically pinocytosis or phagocytosis by macrophages) [7]. They also can go to one or more filtering organ (e.g., kidney or liver) to be excreted. Some, that have a gaseous component, can be removed at least partially through the lungs (e.g., methyl methacrylate monomer from bone cement) [4].

Degradation can also be classified by whether it is mostly a change in mechanical properties or a change in mass (material degradation). Material degradation normally results in loss of mass and can occur from the outside in (bioerodible) or non-uniformly [4,7]. Non-uniformly can be cells growing into a scaffold and breaking down the material (usually with chemicals and enzymes triggered by inflammation) to facilitate tissue ingrowth [7]. This can be helped by having a phase of the implant degrade preferentially. Porous scaffolds can degrade within the pores as well as from outside in. Similarly, metal oxidation (corrosion) can occur inside pores or preferentially (due to composition or differences in local microenvironments) [7].

Mechanical property degradation can parallel the material degradation, if the material is uniformly surface erodible [4,7]. In this case, material properties (modulus or the relationship between stress and strain) typically remain constant, while ones related to cross-sectional area (stiffness or the relationship between load and deformation) will decrease proportional to the loss of material [34]. Material properties (e.g., modulus) are designed to be independent of cross-sectional area to be a property of a given material [5]. It is determined by the amount of force per unit area (cross-sectional area) (called stress) that can expand a sample to twice its original length (100% strain) in order to be independent of sample dimension [4,5]. Where stiffness is how we interpret the relationship between load to compress a spring a certain amount or bend a rod a certain amount.

In some, cases the modulus can change. e.g., porous materials can be considered composites of the material and air (this is a good approximation up until a relatively high porosity) [7]. In this case, the modulus is proportional to the volume fraction of material [7]. Therefore, degradation that makes the material more porous reduces the modulus related to the porosity. Polymers that can lose molecular weight (e.g., breaking of chains hydrolytically) without much mass loss, essentially decrease the modulus [4,7].

It is also possible to reduce the strength of the material (stress that leads to failure). Failure is essentially a crack that goes all the way through the material. Things such as surface irregularities can make cracks easier to form and therefore reduce the load required to break the material, without significantly affecting stiffness [4,34]. Therefore, non-uniform degradation can lead to a reduction in strength properties without affecting modulus [34].

#### 1.4.4. Incorporation into the Biomaterial

##### Drug Delivery

The biomaterial can be used to delivery biological response modifiers [4]. In a degradable system the release is generally triggered by degradation [7]. Assuming the biological response modifier is a growth factor, it can be attached (typically covalently especially for natural biomaterials) to the biomaterial, encapsulated by it, or mixed in with the polymer [4]. If attached, degradation of the biomaterial or the attachment controls the release. Biologic materials tend to be broken down by enzymes from the cells that grow into the scaffold. This can give a release rate controlled by the healing rate (biofeedback control) [4].

If encapsulated, release is diffusion controlled which changes as the biomaterial degrades [7]. If mixed in and the degradation is surface erodible it would mostly be degradation controlled. If mixed in and the degradation is hydrolytic, degradation can occur throughout the biomaterial and there would be some diffusion controlled release as well.

Concerns with growth factor delivery are maintaining activity, release kinetics, and release distribution [7,35,36]. Maintaining activity of growth factors can be difficult, since many lose activity quickly when unprotected *in vivo* [36]. 

Activity of a growth factor is usually dependent on having one or more binding sites maintained and available [7,11,33]. Binding sites are a grouping of amino acids in a specific spatial orientation [7.33]. The amino acids are normally not sequential in the protein sequence of the growth factor. The binding site is created and stabilized by cysteine bonding, folding of the protein chain and possibly the presence of other molecules including another protein chain (two growth factor chains often come together—called dimerizing) [7,11]. Availability of the binding site can be reduced by excretion, blockage of the binding site by another biomolecule, or change in 3D orientation of the chain (to reduce space around the binding site or protection [7,33]. Loss of the 3D orientation of the binding site leading to deactivation is called denaturing and can be due to breakage of bonds in and around the binding site or chemical alteration of amino acids (or other biomolecules) in and around the binding site [7,33]. Changing of the ends of each amino acid can be done with changes in pH (add a hydrogen to the amine side or remove a hydrogen from the acid side) [11,35]. Therefore, small changes in the environment can lead to denaturing or require biochemicals to break bonds (crosslinking bonds or between adjacent amino acids) [11,35]. Since growth factors are easily denatured *in vivo* (half-life measured in hours for many) as well as can be very powerful if released they are normally attached to other biomolecules or intracellular *in vivo* [11,33,36]. Synthetic delivery systems do not always protect the growth factors like they are *in vivo* [11]. 

So activity can be lost during storage, while incorporated in the biomaterial (or while being incorporated), or as part of the degradation process [7,11]. There should be activity assays to check the growth factor activity in each stage [4]. Although biological activity is the most important assay there could be ones to check for reasons for activity loss due to loss of structure by monitoring molecule weight or antibody binding [4,7].

The growth factor once released from the biomaterial can have a local effect, be distributed systemically (typically through the bloodstream), or be sequestered in a specific location (wound site, filtering organ, or be targeted to a specific location or type of cell (e.g., cancer cells)) [4,7,11]. It is also important to make sure the growth factors are presented in a way to produce the desired response. For example, some growth factors are chemoattractants that work by having a gradient (so how it is released is important), some require going inside a cell, some can still be attached to something else, and some require specific concentrations to work as desired (usually a minimum, but some can have a negative feedback- loop) [7,11].

##### Cell Incorporation

Cells can be stimulated *in vitro* to produce extracellular matrix (ECM) to help make the biomaterial they are incorporated into, mixed in with a separate biomaterial, or are injected locally (also systemically and home to the biomaterial at the wound site) [4,7,35,36,37]. Cells can be ones that are native to the tissue where it is to be placed. Cells can also be stem cells that are pushed to differentiate along the specific lineages by factors in a bioreactor (on the biomaterial surface or created in the bioreactor); *in vivo* by factors on or in the biomaterial, injected, are present in the wound, or an external stimuli; or by genetic modification of the stem cells [4,7,38,39,40,41,42,43,44,45,46,47,48,49,50,51,52,53,54].

The main concern with cells are similar to growth factors: maintaining activity [4,7]. With cells, activity is tied to viability or change in phenotype as well as a change in cell activation factors [4,7,33]. Viability is a concern during storage, while inside the biomaterial (or while being incorporated into the biomaterial), or as part of the degradation process. The activity desired can be to populate the tissue defect through differentiation, proliferation, and/or migration; to recruit other cells; to produce ECM; or to produce or induce vascularization [4,7]. Note it is difficult to do all of these at the same time and with the same cell population.

Cells can also serve as growth factor delivery systems. This can be using various stimuli to get the cell to produce the desired growth factor or genetically modifying the cells to overproduce the desired growth factor [4,7,14] (this is one of the studies in this issue).

## 2. Strategies

Previously design options were presented, now how these designs are used will be discussed. First the three general strategies (scaffold, graft, and outside of the defect) of the functional biomaterial will be presented, then some specific applications (predominantly from my research) with design strategies that use the modifications previously mentioned. The emphasis will be at the top of the hierarchy: degradable/regenerative scaffolds (the first general strategy). The third general strategy will be covered for a load-bearing device. 

The specific applications selected are also to help define the field and types of research that can be done. Again, the specific research approaches (including materials and processes used) and design optimization studies are to provide examples of research in BER (particularly ones in this issue), not to suggest these are the best or only approaches to meet the clinical performance constraints. Since all but two of the articles in this issue come from my students, the specific applications are predominantly based on work of my past and current students. The specific approaches used will be justified based on their potential to meet the clinical performance requirements, not on their comparison to other approaches.

From a commercialization standpoint some additional design constraints relate to development cost (including time and ease of regulatory hurdles). This normally means the fewest modifications particularly ones that use biological components. In most cases, the use of these biological compounds can improve the ability of the scaffold to enhance the regenerative response, but may not be needed if the desired clinical performance design constraints can be achieved without them. Unfortunately, clinical studies are needed to prove a design meets the clinical design constraints, but animal models can be good approximations. From a design perspective either the basic strategy is used and modifications (biologics and other biologic response modifiers) are added to achieve the performance design constraints or systems are designed to meet the design constraints and then simplified, while still meeting the design constraints.

### 2.1. General Design Strategies

In general, the functional biomaterial is either a degradable scaffold or a graft substitute. The specific strategies will concentrate on the degradable/regenerative scaffolds, but relate concepts to grafts and graft substitutes. In addition, some devices will work adjacent to the defect or wound to help in the regenerative process and will also be discussed for load bearing applications.

The end result should be the functional biomaterial, if a scaffold, is degraded and totally replaced by new tissue. If it is a graft substitute, it should get incorporated into the native tissue. Ideally, the new tissue should be exactly like the surrounding tissue and the graft substitute should act like the native tissue once healed in. In reality, however, the new tissue formed as well as the graft substitute remodel over time. In fact, most autografts work this way as well [4,7]. Typically, the new tissue formed in a skin defect is predominantly type III collagen that eventually gets remodeled to type I collagen [7].

In current clinical practice, the remodeled tissue ends up with some repair or scar-like qualities versus total regeneration. In this review, the emphasis will be on strategies using degradable/regenerative scaffolds to come close to the ideal with studies on how to move closer to the ideal. Again, although grafts and graft substitutes are the current “gold standard” they still tend to get remodeled over time and act similar to degradable/regenerative scaffolds, particularly at the host–graft interface [4,7].

There are also additional external strategies used to enhance regeneration, which do not directly involve the biomaterial, but are benefitted from the strategies described in this section. This would include strategies that clear up an infection prior to using the implant, can control the degradation rate, or can alter cell activity (differentiation, migration, proliferation, ECM production, etc.). 

Infection control can include the biomaterial: antibiotic release, modification of the biomaterial surface to prevent bacterial attachment (anti-fouling materials as well as metals like silver and copper) [4]. The main goal is the prevention of biofilm formation on the biomaterial, which protects bacteria from antibiotics and formation of a biofilm. Once a biofilm forms it is difficult to remove and usually requires removal of the implant [4,7]. An infection (high concentration of bacteria in the tissue) will usually not only prevent healing (causing necrosis in many cases), but also can increase degradation rate [7]. Biofilm formation is typically not an issue with degradable/regenerative materials, since degradation of the material helps break up any biofilms that have formed. Infection can still be a clinical problem, if the rate of surface erosion is too slow or the defect has a high but sub-clinical level of bacteria [7]. For example, graft substitutes and scaffolds are not used for pressure ulcers due to their relatively high bacteria burden [7,55,56,57].

Increasing degradation rate can also be an external control of infection. External degradation control can be done by adding chemicals or other catalysts to the system as well as adding energy to the system (e.g., heat, ultrasound, or mechanical loading) [7]. 

Including in strategies to alter cell activity are cells and biochemicals added around the implant or systemically that can home to the wound site (which were mentioned in the respective modification sections) [7]. Most of the other strategies are similar to degradation control requiring adding energy to the system (e.g., heat, ultrasound, electric fields, and magnetic fields), which includes mechanical loading (either constant or cyclic), e.g., vacuum assisted closure [7].

The need for modifications or added bioactivity to the biomaterial is dependent on the clinical performance design constraints. Although, in this review, the emphasis is on strategies to enhance the regenerative response, for commercialization, the design constraints of development cost and profitability limit the options. Although the focus will not be on commercializable strategies, the designs discussed for skin regeneration were selected with development cost and profitability in mind [4,7]. Treatments were designed to be at the patient’s home (particularly for the spinal cord injured with pressure ulcers—who have difficulty traveling) to be able to reduce costs of treatment. In addition, biologics selected were either already available or could come from the patient to make the regulatory path easier.

#### 2.1.1. Degradable/Regenerative Scaffolds

There are a number of different ways to design scaffolds to degrade and regenerate. For example, the implant can be surface erodible and broken down enzymatically as the tissue front moves forward. To truly serve as a scaffold and enhance the regenerative response, the implant should have pores (channels or spaces between fibers) in which the cells can grow into, attach to the biomaterial, and produce ECM [4,7]. Note for fibers, the pores are essentially the space between the fibers [31]. The pores can be designed into the system initially or pores can form due to preferential degradation of a phase of the implant. The scaffold serves to enhance the regenerative response by allowing cells to grow into the biomaterial, attach, and produce ECM versus having to produce ECM first in order to grow into the wound [7]. 

This is similar to the formation of a fibrin clot as the provisional matrix (degradable scaffold) in many wounds [11]. In this case the fibrin fibers serve as the degradable scaffold on which cells can move along, attach to, and produce ECM [11]. In a full-thickness skin defect without a provisional matrix, the fibroblasts have to produce the ECM before they can migrate into the wound space [11]. This requires blood supply to be within 100 μm of the fibroblasts to provide enough oxygen (30–40 mmHg) for the fibroblasts to produce the ECM [7]. Therefore, migration into the wound space is similar for ECM, fibroblasts, and blood vessels. The oxygen requirements for fibroblast viability and migration are lower than to produce ECM so with a scaffold the fibroblasts can be more than 100 μm ahead of the blood supply and the blood supply does not have to wait on fibroblasts to produce ECM [7].

The new tissue can also form from the inside out. This normally requires seeded cells to produce the ECM inside the biomaterial and may require a vascular supply to be formed first (also requiring cells—e.g., endothelial progenitor cells) [7,54,58]. Some use co-culture of endothelial cells (or progenitor cells) with fibroblasts (or stem cells) for vasculogenesis (blood vessels formed from the inside out) [7]. Again, the pore structure can be designed into the system or formed as the biomaterial is preferentially degraded away (e.g., hydrolytically or by the cells).

#### 2.1.2. Synthetic Graft

As previously mentioned the newly regenerated tissue in the skin wound or defect is more similar to a pedicle graft than a free graft. The new tissue, however, typically is incorporated into the surrounding tissue and has a good blood supply connected to the surrounding tissue all the way around. A pedicle graft will have a blood supply, put not connected all the way around and therefore still has interfaces that must heal. Even pedicle grafts typically get remodeled after healing-in, just like the newly regenerated tissue [7]. 

Therefore, the biomaterial essentially forms a graft once it is degraded. As the initial structure of the biomaterial comes closer to the native tissue and is populated by resident cells it can behave more and more like a graft itself (but a free graft without blood vessel attachment to surrounding tissue or likely no blood vessels at all) with less and less degradation, regeneration, and remodeling required [7,22,23,24,25,26,27,28,29].

#### 2.1.3. Devices Adjacent to the Defect or Wound

In this case, it is external to the defect and may or may not be outside the body. To a certain extent any skin wound dressing can protect the wound and in some cases keep it moist, to enhance the healing response. To really be considered in the biomaterial enhanced regeneration category it should be more active like delivering a factor into the wound. For bone, a fracture fixation device that degrades over time giving more and more load to the fracture as it degrades would also qualify.

### 2.2. Specific Strategies

This section will review some specific strategies for degradable/regenerative systems for full-thickness skin defects or wounds. In addition, the use of degradable systems in load bearing applications will also be discussed. Although there are many types of load bearing devices, examples will be confined mostly to fracture fixation of long bones with degradable systems. Skin devices will focus on tissue adhesive scaffolds, with fracture fixation emphasizing degradable metals.

#### 2.2.1. Skin Scaffolds

My research has focussed on both optimizing implant bioactivity as well as its scaffolding ability. In biomaterial enhanced regeneration they are synergistic, with the bioactivity being enhanced by the scaffold and the scaffolding properties being enhanced by the bioactivity. For optimal bioactivity, both environmental changes (oxygen and electromagnetic fields) and biochemical modifications (growth factors and cells) have been assessed *in vitro* and *in vivo* in order to optimize the regenerative response. For optimizing the scaffold, different materials with different configurations, degradation rates and drug delivery kinetics have been assessed *in vitro* and *in vivo*. The ultimate goal has been to design systems suitable for the treatment of both pressure ulcers and burns that could be used in open wounds as well as in conjunction with skin grafts.

##### Bioactivity

Critical to determining the appropriate system are the rates of migration of the key cells. Fibroblasts migrate up to 200 μm/day [9,10], but will only produce ECM if the blood vessels and nutrients are within 100 μm. Angiogenesis for a 0.5 mm collagen/GAG system takes 7–9 days [9,10] (50–70 μm/day). This slows the fibroblast ingrowth across 500 μm to 5–8 days (60–100 μm/day) versus 2.5 days (200 μm/day). Therefore, angiogenesis becomes the rate-limiting step.

Angiogenesis is also important to support the epidermal layer. Epidermis migrates at about 0.33 mm/week over viable vascularized dermis, but only half that if it has burrow through tissue to find viable tissue [59]. Therefore, the speed in which vessels permeate the scaffold determines the earliest that the matrix can support epidermal cells (at least a week for the 500 μm collagen/GAG template).

Angiogenesis is also the key in reducing the risk of infection in the graft because it can bring cells for an acute inflammatory response to kill bacteria [7,59,60]. Researchers have tried to circumvent the slow angiogenesis by providing a cell culture like medium for the cells (with antibiotics) [61]. This may not be logistically possible in all cases plus could be avoided with a different design [7].

Therefore, strategies to speed up angiogenesis are needed. This would include use of angiogenic growth factors such as fibroblast growth factor FGF [7]. Additionally, modification of the matrix configuration, such as optimizing the porosity or making the matrix less than 200 μm thick, could assure that angiogenesis was not the rate-limiting step. Further vasculogenic cells (e.g., endothelial progenitor cells) can be used around the implant, inside the implant, or injected systemically (and home to the wound) [58]. These cells can help the formation of blood vessels both from outside in (angiogenesis) and inside out (vasculogenesis).

With natural biomaterials, such as albumin or fibrin, delivery of a biological response modifier can be accomplished in a number of different ways [11]. The biological response modifier can be impregnated within the matrix, attached to the polymer chain, or included through intrafibril entrapment [11,62]. In addition, seeded cells (possibly genetically modified) can be growth factor drug delivery systems. Incorporation during the polymerization process is similar to intrafibril entrapment. In this case, if the substance is larger than the intrafibril pores, it is released only when the natural material degrades [11,62,63,64]. Therefore, the release is controlled by the rate of phagocytic cellular infiltration, and thus under biofeedback control. Additionally, the degradation is at the wound edge and thus gives the appropriate gradient to stimulate further angiogenesis and tissue healing [7].

The use of a degradable matrix to deliver a biological response modifier, such as a growth factor, can protect the growth factor until release, since growth factors typically have a short half-life *in vivo* [11]. The short half-life has potentially been a problem in clinical studies, leading to reduced efficacy, and/or increased expense associated with daily administration [11]. Thus, a natural polymeric matrix such as fibrin or albumin, can protect the biological response modifier until release, serve as a biofeedback controlled drug delivery system, and provide an adherent tissue scaffold during healing [11].

Growth factors are needed, if the clinical performance design constraints cannot be met without them. In this case, growth factors are used primarily to enhance tissue ingrowth by speeding up the rate limiting step of angiogenesis [4,7,11,35,36]. 

Again, cells can be injected locally or systemically as well as be seeded into the scaffold [11,39,49,53,54,58]. All three have been used with tissue adhesives [11]. For skin, cells used have been keratinocytes and stem cells for connective tissue (mesenchymal stem cells) and blood vessels (endothelial progenitor cells) [4,11,58]. In one study (included in this issue), mesenchymal stem cells were also genetically modified to over produce a regenerative growth factor [54]. 

Again, issues with using cells are related to viability and activity (including cell–matrix interactions). This is mostly an issue for cells incorporated into the scaffold. Tissue adhesives used have two components that are mixed in the wound. Studies need to be done to determine which components the cells are to be stored with as well as how activity is maintained throughout the production, delivery, and time *in vivo*. Typically, the cells are used to enhance regeneration by both increasing blood supply and tissue formation (mostly ECM production) [7,11].

Although most cells in seeded clinical skin systems have a limited lifetime and serve mostly as growth factor delivery systems, it is important to determine how much of the intended role the cells play [7,37]. Since these cells are typically available *in vivo* it is important to make sure the injected or incorporated cells (at higher concentrations than available *in vivo*) are more effective than trying to increase recruitment of these cells. The ability to recruit stem cells to a wound site usually decreases as we age as well as in certain pathologies such as diabetes [7]. Again, the cells are only needed, if the clinical performance design constraints cannot be met without them.

In order to determine the optimal level of oxygen needed for healing, a series of *in vitro* and *in vivo* studies were done. *In vitro*, oxygen at 20% O_2_ (160 mmHg) [65,66] was found to stimulate the greatest increase in fibroblast activity with a concomitant decrease in macrophage activity. In an *in vivo* study, both the oxygen level and oxygen gradient were modified, based on preceding *in vitro* studies, to help determine the optimal clinical oxygen treatment protocol [67,68]. Oxygen treatment, corresponding to the 160 mmHg *in vitro* level (70%), significantly accelerated the healing response with a more occlusive (oxygen impermeable) wound dressing, in the early healing stages. A more oxygen permeable wound dressing however, provided the better cellular and tissue response at the later healing stages [67,68]. A further study, examined a lower oxygen dosage (40%), which is closer to the more clinically acceptable 6 liters per minute, and found a similar acceleration in the healing response [69]. It appears, therefore, that the oxygen gradient is only helpful in the early inflammatory stages when macrophages are present, until the granulation tissue is formed and optimum oxygen levels can be achieved without hyperbaric oxygen.

The use of low-frequency pulsating electromagnetic fields (PEMFs) was also examined to more fully understand its effects in the treatment of full-thickness defects, in a rabbit model [70,71,72,73]. It was found that a magnetic field of 2–2.8 mT at a frequency of 75 HZ applied for 240 min daily for one week, significantly accelerated the healing response [70,71]. An additional *in vivo* study was done to determine the optimum parameters for the PEMFs to be implemented for soft tissue regeneration and overall wound healing [72,73]. This study (included in this issue), demonstrated that although PEMF accelerates the healing response in all cases, specific combinations of frequency and intensity levels produce a specific cellular response [72,73]. It is possible that the optimal PEMF system may involve a series of different frequencies and intensities at various stages of the healing process [72,73].

Optimizing the electrical field for three types of skin cells (keratinocytes, fibroblasts, and endothelial cells) was also done, *in vitro* [74,75,76]. Using electrical field gradients over a range from 100–300 mV/mm (similar to the electrical fields seen *in vivo* during wound repair or stimulated by a clinical device evaluated), up-regulation of gene expression (using micro-arrays) and production of specific biochemicals (using real-time polymerase chain reaction (RT-PCR)) was done [76]. Although 100 s of genes were up-regulated or down-regulated for each cell type, there were eleven or less significantly altered for each cell type [74,75,76].

Although not a molecular switch for production of individual biochemicals there are specific windows of field characteristics that lead to maximum induction of critical genes for each cell type [7,74,75,76]. Therefore, it appears that the normal change in the electrical field during wound healing as well as altered exogenously has a significant effect on the cell activity of key skin cells [7,74,75,76].

*In vitro* growth factor studies have been done with PDGF, FGF-1, and TGF-β with or without collagen, PLA or fibrin substrates [4,7]. Although maximum values were found in the nanogram/ml range (optimal fibroblast proliferation with and without PLA and collagen implants) [65,66], *in vivo*, these levels showed no significant effect [77].

Therefore, for *in vivo* studies increased growth factor concentrations were evaluated [7]. An *in vivo* study was done to compare the effectiveness of TGF-β and FGF-1 for the treatment of full thickness wounds created on the dorsum of New Zealand white rabbits [78,79,80]. Each animal had a control (no treatment), TGF-β (2 μg/cm^2^) incorporated in a collagen matrix, plain collagen, and collagen with added FGF-1 (100 μg/cm^2^) [78,79,80]. Even though growth factors were incorporated into the matrices, the total release was so quick (a few days), it was actually more similar to a topical dose than a controlled release system [78,79,80,81,82,83,84,85,86,87,88,89]. Although the TGF-β incorporated matrix showed enhanced angiogenesis, it was concluded the wounds treated with the topical FGF-1 with the collagen matrix healed slightly better overall [58,81].

For cell studies, both mesenchymal stem cells and endothelial progenitor cells have been used [54,58]. They have been incorporated into albumin tissue adhesive scaffolds as well as injected locally or systemically to help heal full thickness skin defects [54,58]. The mesenchymal stem cells were also genetically modified to over produce a regenerative growth factor (TGF-β_3_) [54]. For the mesenchymal stem cells the combination of incorporation and local injection had the biggest effect on healing with almost a doubling of the epithelialization rate for covering the wound after the first week [54]. For the endothelial progenitor cells the combination of incorporation and systemically injected had the biggest effect on healing after two weeks [58]. It appeared that the systemically injected cells didn’t have a significant effect until the second week [58]. Also they seemed to enhance the blood supply more inside the scaffold than at the periphery [58]. 

##### Scaffold Materials

To optimize the scaffold, different materials have been evaluated, including collagen, PLA, fibrin, and albumin [77,78,79,80,81,82,83,84,85,86,87,88,89,90,91,92,93,94,95,96,97,98,99,100,101,102]. Tissue adhesives (fibrin and albumin) have been the major focus, since they can set up in situ, filling voids and irregular shapes, but can also have growth factors or cells incorporated at the time of polymerization [4,7]. In addition, the ability to tie the drug delivery and degradation to cellular infiltration establishes a biofeedback system that is tailored to the individual patient’s healing rate [7,11,94]. As previously mentioned the use of a scaffold can change the migration rates of the cells and tissue, since the fibroblasts do not need to produce as much ECM.

Fibrin is derived from fibrinogen in blood. As a tissue adhesive, it is generally supplied as a two-component kit consisting of human fibrinogen/Factor XIII and bovine thrombin/CaCl_2_. These fibrin sealants have been used since 1972 in Europe where a commercial version was available, and later in the U.S. [11]. Until almost 2000, studies in the U.S. used only autologous or single donor preparations [4,11,84,85]. Clinically, the fibrin matrix has been used as a hemostatic agent, for tissue anastomosis, as a fluid barrier, as a drug delivery vehicle, as a tissue scaffold, and as a matrix for cultured keratinocytes [62,63,82]. Fibrin sealant used for skin grafting has been shown to increase strength of attachment to the wound bed compared with staples [85,100,101,102,103], leading to less seroma formation [85], and wound contraction [104]. Making the fibrin porous allows for quicker graft take by providing a scaffold for the blood vessels to grow through, without the matrix having to be broken down first [7]. In one study (included in this issue), however, as fibrin was made porous it appeared that the shear strength was inadequate to handle physiological loading [105].

In full-thickness defects, a degradable fibrin scaffold has been shown to increase the angiogenic and tissue response over controls [88]. Apart from the scaffolding effect, these degradable systems demonstrate other desirable characteristics. Studies have also shown the utility of fibrin as a degradable adhesive for both blood vessel anastomosis [106,107] as well as skin graft attachment [85]. The concentration of fibrinogen has been varied between 15 and 70 mg/mL in an effort to determine minimum setting time and maximum adhesive strength. It was found that the strength of the fibrin clot increased linearly [85,100,107] while degradation rate decreased [103,104] with increasing concentration. In addition, near maximum strength was achieved in the first few minutes [85,100,107]. Interestingly the commercial fibrin glue (Tissel^R^ or Tissucol^R^) made by a modified cryoprecipitation technique is not as strong as autologous cryoprecipitate preparations, made at UAB (due to its slightly lower concentration) and attains full strength at a slower rate) [85,100]. Also platelet rich plasma can be used and mimics low concentration fibrin glue [86].

While fibrinogen can be treated with detergents and organic solvents to kill enveloped viruses, such as HIV-1 and hepatitis B, the process does not inactivate viruses that lack lipid envelopes, such as hepatitis-A and human parvovirus B19 [108]. This was one of the major reasons that the use of pooled fibrinogen took so long to be approved by the FDA, and only for hemostasis applications [11]. At this time, in other clinical applications, therefore, fibrin had to be extracted from the blood or plasma of the patient [85]. This required a lag time, between blood or plasma extraction, and availability of the fibrinogen for surgical use, as well as the need to take blood from a patient whose health was already compromised [85].

The concerns and logistical difficulties with fibrin glue have provided impetus to look at other tissue adhesives. Currently, an albumin system is being evaluated as regenerative tissue adhesive scaffold [11]. Albumin, while also a blood product, can be processed at high enough temperatures to inactivate viruses that are potential problems in fibrin systems [11]. Albumin is widely used and accepted by the medical community, and has been approved by the FDA for clinical use [11]. In addition, because albumin is derived from pooled human plasma, it is a more consistent product than autologous fibrin [11]. It is also more convenient for the same reason, with no advance blood donation and processing prior to clinical use as was the case of fibrin. Further, adhesive albumin, such as that produced by crosslinking with poly(ethylene glycol), has been shown to possess mechanical properties superior to both autologous and commercially available fibrin glues [109].

Human serum albumin is the most prevalent soluble protein in blood [92,93]. The linear pattern of loops of the 66 kDa polypeptide, with short-range coupling between half-cystines, provides for both flexibility of the albumin molecule and resistance to harsh conditions [11]. The loops can associate, forming a globular structure, or can separate reversibly [11].

Since World War II, albumin has been widely used in circulatory therapy, primarily for circulatory support during shock [11]. Polymerized microaggregates of albumin can serve as agents for radiocontrast and ultrasonic imaging of the circulatory system [11]. Albumin is also used to coat prosthetic blood vessel grafts and vascular catheters to passivate these surfaces so as to minimize platelet aggregation and thrombotic consequences [11,110,111,112,113,114].

The use of albumin as a medical adhesive is relatively new [11]. It must be cross-linked to achieve the necessary strength, but could be used in the same applications as fibrin: hemostasis and wound closure [92,93].

Disuccinate cross-linked albumin has also been shown to inhibit bacterial growth on titanium (cp-Ti) surfaces [11,114,115] as well as effectively deliver gentamicin and increase the half-life of the antibiotic [116]. This is a significant advantage over fibrin, which is claimed to accelerate infection [11]. Because it is a natural human protein, tissue proteinases will degrade albumin [92,93]. Thus, in a healing wound, crosslinked albumin adhesive should degrade as the healing tissue advances [92,93].

In one study [109], a solution of 25% (w/v) human albumin was mixed with modified-PEG (mod-PEG), to form solutions with either 20% or 5% (w/v) final PEG concentration. Both adhesive albumin systems were tested on samples of skin (skin–skin), by imposing a shear force until failure, using an Instron mechanical testing machine [109]. Results were compared to simultaneous tests using fibrin adhesive. The 25% albumin-20% mod-PEG adhesive albumin system was also tested between aluminum samples (Al-Al, no skin) [109].

The highest shear strength, obtained with the 25% albumin-20% mod-PEG system, was approximately 5 times that of the fibrin glue. The 25% albumin-5% mod-PEG system shear strength was approximately 1.5 times stronger than that of the fibrin system. Shear strength of the 25% albumin-5% mod-PEG system was comparable for both the skin–skin and Al–Al tests [62].

In other studies, albumin glue, fibrin glue, cyanoacrylate, or sutures were used to close incisional wounds on the dorsum of rats and rabbits [4,11]. Wounds closed with albumin and fibrin had excellent healing, with no inflammatory reaction observed histologically [4]. There was a minimal lymphocytic reaction in the sites treated with albumin [4]. In the wounds closed with silk sutures, there was complete healing, but with a more prominent scar than in the albumin and fibrin treated wounds, and some foreign body reaction around the sutures [4,11]. In the wounds closed with cyanoacrylate, there were ulcerations, necrosis and a severe inflammatory reaction observed at the wound site [4]

Additionally, it was found that the incisional strength for an albumin adhesive could be as strong or stronger than sutured wounds after one and two weeks *in vivo* as well as significantly stronger than the fibrin adhesive both initially and after one week [109]. Interestingly at high albumin concentration the adhesive does not show the twofold increase in incisional strength, between the first and second week, seen in sutures and other albumin glues, possibly due to too slow a degradation rate [4,11].

Further studies were done to optimize the albumin system as a regenerative scaffold (part of these studies are included in this issue). This included: different functional groups on the PEG cross-linker, different porosities, and different amounts of albumin [4,11]. Also, the effect of PEG crosslinking on FGF-1 activity [92,93,94,95,96,97,99]. This allowed selection of the albumin systems, which best matched the design constraints for a specific application [4].

##### System Design

Based on previous work, FGF-1 was selected as the angiogenic agent for skin applications. Both *in vitro* and *in vivo* studies have indicated that the tissue response is dose dependent and a maximal response is reached at an intermediate dose [88].

Fibrin was selected for its adhesive properties [4,11,82] as well as its ability to serve as a degradable drug delivery system for wound healing [88,89,90]. Again, the degradation rate and adhesive strength can be controlled by fibrinogen concentration [11,54]. Additionally, *in vivo* and clinical studies have been done to test the use of autologous single and donor fibrin matrix for skin graft attachment in burn patients (included in this issue) [85,98,117]. It was found that the fibrin hemostasis and early graft adherence led to an excellent graft take with reduced scarring [85,98,117]. When compared to conventional treatments, this technique led to shorter hospital stays, minimal postoperative care and immobilization, no pressure dressings, and prompt start of ambulation and physical therapy with an early return to normal activities [85,98].

For a drug delivery system, there was a concern due to the short biological half-life of FGF-1. This would require that the FGF-1 be protected within the matrix. In the case of fibrin, it has been shown to covalently bind with FGF-1 [94,118]. Additionally, immunolocalization studies have indicated that there is a uniform distribution of FGF-1 in the fibrin matrix [4,11]. Release studies using FGF and fibrin have indicated that although there is an initial high release rate of about 30%, the subsequent release rate is relatively constant and proportional to the degradation rate [88,119]. To help show *in vivo* activity, a comparable enhancement of wound healing was seen with a topically applied dose, designed to mimic the fibrin/FGF-1 release kinetics, compared with the fibrin/FGF-1 system [90,119].

Although the fibrin matrix, due to its own biological activity serves as a reasonable scaffold, better scaffolds can potentially be made by optimizing the configuration as well as the bioactivity [4,11,120]. In a rabbit ear ulcer model, full-thickness defects were treated with the fibrin matrices in two different pore configurations [90,119]. The more porous implant (modified fibrin) showed increased angiogenic response [90,119]. The levels of porosity and pore size can be optimized for various applications. Even in unoptimized systems, FGF-1 in a non-porous fibrin matrix was capable of complete epidermal regeneration with dermal filling of full-thickness defect and minimal contraction (20%) within two weeks, while controls took at least three weeks to heal and healed mostly by contraction [91].

For skin grafts, it was anticipated that making the fibrin porous would allow for quicker graft take by providing a scaffold for the blood vessels to grow through, without the matrix having to be broken down first. Again, in one study, however, as fibrin was made porous it appeared that the shear strength was inadequate to handle physiological loading [58]. It is also possible that using a thin adhesive layer (200 μm or less) would obviate the need for a porous structure as seen in a clinical study [98,105].

Overall, the fibrin/FGF-1 system [4,11] when used in open wounds has led to the best overall healing, compared with all other treatments, with complete epithelialization and minimal contraction. This is likely due to the increased angiogenesis, mostly due to the FGF-1 as well as the increase in new tissue formation due to the fibrin scaffold [11]. Similarly, for meshed skin grafts, FGF-1 incorporated into a fibrin has led to the best graft healing [3]. The FGF-1 concentration that has worked best overall is 10 μg/mL, which when spread in a 200 μm layer is 10 μg/ 50 cm^2^ [98].

Studies have shown that the albumin system can perform as well or better than the fibrin systems in terms of strength and tissue response *in vitro* and *in vivo* [4,11], but has not been evaluated clinically yet. The system has been optimized for meshed skin graft attachment, incision wounds, and full-thickness defects [4,11].

In full thickness defects, albumin scaffolds have worked best when used in conjunction with mesenchymal stem cells [54]. Since there is both hydrolytic and enzymatic degradation for an albumin system, with the hydrolytic degradation controlled by the functional groups on the PEG, there are more options available than for the fibrin systems (study included in this issue) [92]. Porosity is also another way to control degradation rate. With the systems tested, it was found that the porous systems worked as scaffolds by allowing ingrowth throughout the scaffold by one-week. However, by two weeks the pore structure, ingrowth, and epithelialization were similar independent of porosity and pore architecture. [11,105].

The only clinical studies I have done are with the fibrin/FGF-1 system in burns. For one study, a meshed skin graft was broken into three groups and was attached with: (1) fibrin with FGF-1, (2) fibrin without FGF-1, or (3) a stapled control [98]. This study is presented as part of a paper included in this issue. Another study, was to look at the advantages of using fibrin over staples for burns and other skin defects [85].

A critical part of each study has been the development of non-invasive clinical assessment tools to measure the effectiveness of the treatments [83]. The three main variables for comparison are healing rate, angiogenesis, and tissue stiffness [83]. These assessments are done weekly for the first few months and monthly thereafter. For healing rate, the epithelialization rate and contraction rate are determined in a manner that gives the rate independent of wound size [83]. The angiogenesis is determined by a scanning laser doppler, which gives a 2-D map of the blood perfusion [83].

**Figure 3:**
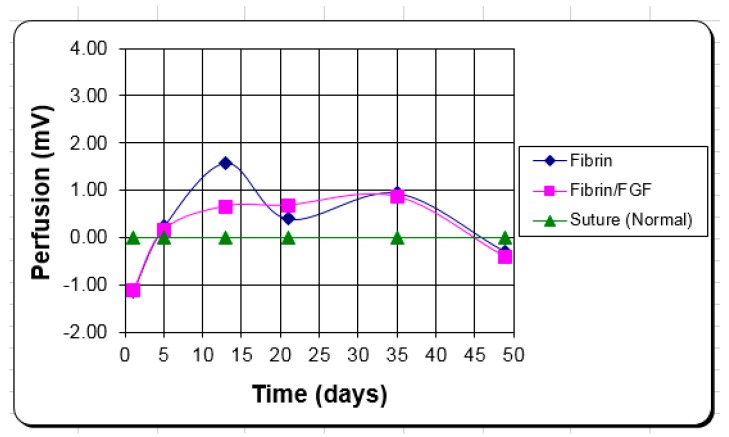
The clinical blood perfusion changes over time for meshed skin grafts adhered with fibrin (with and without FGF-1) or sutured into place in the same patient. Perfusion level was normalized to the sutured control for each graft.

Results indicated that the healing rate was higher for both fibrin treatments and essentially showed a 5-day lag with the fibrin systems healing within 16 days versus 21 days for the controls [98]. The tissue stiffness was also comparable to normal skin versus about twice the stiffness for the controls [98]. The blood perfusion level was also higher for the fibrin systems (Figure 3) [98].

Figure 3 shows the average blood perfusion level for the two fibrin systems for each meshed skin graft over 50 days [98]. In each patient, one skin graft was broken into 3 parts: sutured control, adhered with fibrin, and adhered with fibrin with FGF-1 [98]. In Figure 3, the treatments were normalized to the sutured control for each graft. The blood perfusion level was higher for the two fibrin systems from day 5 until day 45 [98]. This was during the healing phase and the beginning of the remodeling phase [98]. The increased perfusion probably was a big factor in the faster healing and reduction in scarring seen for these meshed skin grafts [11,98].

#### 2.2.2. Load Bearing Systems

Although scaffold design for bone is similar to skin systems a major difference is the mechanical demands. This section will focus on the role of mechanical loading on healing, for a degradable/regenerative system in a load bearing application. Since there are some differences depending on the application due to differences in magnitude and type of loading, one application will be used for illustration: fracture fixation in a long bone using a degradable bone plate. This will be a system adjacent to the defect and therefore not serve as a scaffold. It, however, will be a degradable system that enhances the regeneration process due to its degradation. In this case, the strategy is to transfer as much load to the healing bone as it can handle during the healing process, which should be the guiding principal in virtually all load bearing applications [8].

For mechanical loading the biomaterial actually has two major interrelated functions to help enhance the bone regeneration process [84]:

1. How to stabilize the fracture site (bone to bone interfaces) to allow healing;
2. To control loading to the fracture site to stimulate healing.

The two are related since too much loading (2) will cause a rebreak undermining (1) [8]. Similarly, too much stability (1) will slow healing [8]. The biomaterial controls both by its stiffness, which is based on both the material used (its modulus) and its geometry [7]. Stiffer materials increase the stability of fracture sites, but also reduce the loading placed on the fracture site [7]. A degradable system can reduce the stiffness over time by changing the geometry over time [7].

##### Load-Bearing Implant Design

For a degradable/regenerative system, a good strategy is for the design to provide the needed internal fixation (stability of attachment) while promoting healing for a specified amount of time (depending on the type of injury and the patient’s healing rate) and then degrade away [8]. This would eliminate the need to remove the device, leaving a functional repair that is as close to the original structure as possible [8]. This would require not only healing while the device is in place, but also healing to replace any voids created as it degrades. Also, if the system (bone plus device) early on (and throughout healing) can handle physiological loading then rehabilitation can be started early in the process and be completed sooner [8,34].

The key bioprocess rates are implant degradation and fracture healing [7]. Both of these affect the clinical performance requirement of handling physiological loads early and throughout the healing period [7]. The load handling ability is determined by the properties of the bone/device composite, which change as the implant degrades and the bone fracture heals [7]. Again, the healing rate is affected by the amount of load placed on the fracture, which also changes as the implant degrades and the bone fracture heals [7].

From a design perspective, the stiffer a component is, the higher the percent of the load it is responsible for [7]. Choosing a material that has a modulus (stiffness is directly proportional to modulus) closer to bone (clinically used metal implants have 10–20 times higher modulus than bone) as well as degrading away both serve to increase the loading on the bone (stiffness is also directly proportional to cross-sectional area) [7,16,121].

Although healing rate is the bioprocess, the important parameter is recovery of mechanical properties. Mechanical recovery can be recovery of stiffness, deformation to failure, or load to failure; with the importance of each application dependent [121].

The bioprocess of implant degradation can be used in Figure 2 to determine the change in mechanical properties of the bone plate over time [7]. Again, both the stiffness and the load are proportional to the cross-sectional area [7]. Although it works slightly different for different types of loading (axial, bending, and torsion), axial loading (which can be tension or compression) as is modeled in Figure 2 will show the relationships between implant degradation, fracture healing, and mechanical properties [7]. For simplicity, the model assumes a rectangular cross-section and that the bone plate degrades from the surface inward (erodible). It obviously is more complex with a typical bone plate that is curved and has holes filled with screws, but is still a good approximation [7].

Therefore, a key input parameter is the degradation rate of the bone plate, which determines its mechanical properties over time [7]. Based on previous studies, materials used degraded too quickly for most orthopedic applications [34]. Even if the properties were close to the desired values initially they degraded too quickly for most applications [34]. Polymers and polymer composites also tend to degrade from inside out [8]. Even degradable metals being used for orthopedic applications (Mg alloys) tend to degrade too fast, loosing stiffness too quickly and thus transferring more load to the fracture site than it can handle [122,123].

Although the mechanical properties of the components in the system determine whether the clinical performance design constraints are met, they are controlled by the bioprocess rates [7]. Although the critical mechanical properties are stiffness, deformation to failure, and load to failure these are based on the material properties of modulus, strain to failure, and ultimate tensile strength [7]. In this simple model with axial loading, the critical mechanical properties are related to the material properties by the cross-sectional area [7]. The material properties are independent of size or shape of the device [7]. So, as the cross-sectional area increases the stiffness and load to failure increase while the deformation to failure decreases [7]. 

The critical material properties are therefore modulus and ultimate tensile strength. The simple model in Figure 2 will determine the load and deformation on each of the three components over time, if the properties of the fracture site is determined over time, for a given load [7]. To use the model, the physiological load to be expected needs to be inputted [7]. The critical outputs are the load on the bone plate and fracture site over time as well as the deformation at the fracture site over time [7]. The design goal is to maximize the loading on the fracture site while not exceeding the deformation that would cause failure [7,8]. It is also important that the ultimate tensile strength of the bone plate is not exceeded at any point during healing [7]. Design parameters can be modified to either see how close the system is to meeting the clinical performance constraints or what changes would need to be done to meet the constraints [7]. Again, the healing rate and therefore change in mechanical properties over time of the fracture site is affected by the loading so this becomes an iterative process, which needs to be validated *in vivo* [7]. 

Specifically, for the model of springs in parallel, the deformation is equivalent for the bone plate and the bone (which includes the fracture) [7]. Since stiffness is the ratio of deformation to load, the stiffer material takes up more of the load [7]. With springs in series (the bone and fracture) the load is constant and the deformation is inversely related to the stiffness [7]. Therefore, as the fracture heals it becomes stiffer and more load is transferred from the plate to the bone (including the fracture) [7]. As the plate degrades it becomes less stiff (because it is thinner) and also transfers more load to the bone [34,121]. 

Therefore, with the goal to put as much load on the fracture site without exceeding the deformation for failure, the model for a bone plate needs to include the rate of increase in stiffness and decrease in deformation of the fracture site over time [7]. Again, this will change as the stiffness of the plate changes [7]. 

##### Strategy Example

One specific strategy for long bone fracture fixation was to use a Mg alloy with a surface treatment to slow the degradation. In this case, the Mg alloy was selected since it has a modulus closer to bone than other metallic materials used in orthopedics with a slightly lower ultimate tensile strength [34,121]. The benefit of the surface treatment is that it increases the fatigue strength (load it can handle under repeated on and off loading) and it creates a thin surface layer that can prevent a change in mechanical properties within the time frame of bone healing 3 weeks to 6 months [34,121].

The material properties are fixed, but the surface treatment and the resultant degradation rate alter the inputs into the model in Figure 2. The surface erosion rate would be dependent on the treatment and would be at the slower rate until the surface layer (about 100 μm) is removed. It would then degrade at the faster rate of the base metal [7,121].

## 3. Current Clinical Devices

The goal for this section is to be comprehensive on strategies without covering all the biomaterials used. This is to look at where we are currently and the limitations of these techniques. The emphasis will be on degradable/regenerative systems, but also many of the current other treatments will be covered as well. There also is a future directions section. The first part of the review covered all the possible degradable/regenerative functional biomaterial strategies with specific strategies I have worked on. This section looks at where we are in current clinical practice at the beginning of the 21st century and what approaches are still being developed.

### 3.1. Current Biomaterial Enhanced Regeneration Techniques for Skin

As previously mentioned, there are a number of different types of treatment for skin wounds that use biomaterials. These are in general wound dressings, grafts, and scaffolds. Most of the wound dressings are just to replace the barrier function of the skin. There are however, ones that deliver bioactive chemicals or cells to the wound. Grafts can be made of natural materials or synthetic. In either case, they tend to be replaced and act more like scaffold systems. Although clinically useable devices will change with more and better options available over time, there has not been a big change since the beginning of the 21st century [7,17,18].

There are also a number of biological response modifiers (e.g., growth factors, cells, electrical stimulation) that can be used with the grafts [7,17,18]. The emphasis again in this review is the biomaterial rather than the cells or biological response modifiers.

#### 3.1.1. Grafts (Skin Substitutes)

Most of these grafts have to be remodeled after they heal in and actually serve like a scaffold [4]. Some degrade quickly and essentially serve to be drug delivery systems of growth factors and/or cells) [4].

##### Natural Grafts

Grafts have been used from the individual (autografts), cadavers (allografts), and animals (xenografts) [4]. These have met with varying degrees of success with autografts (free-flaps or pedicle grafts—with and without attachment to the blood supply) the gold standard for most skin wounds, including burns and skin ulcers [4]. These grafts are not included in biomaterial enhanced regeneration unless they are modified in some way.

##### Synthetic Grafts

Although the emphasis is on degradable/regenerative scaffolds, some of the other commercial products will be mentioned. These synthetic materials used are nylon (Biobrane^®^), collagen/GAG (Integra^®^), or decellularized dermis (Alloderm, Graftjacket, and GammaGraft) [4,24]. Biobrane^®^ uses the nylon as the dermis and a silicone membrane as an epidermis implanted in porcine collagen [4,17,18]. Integra^®^ has a “dermal” layer made of bovine collagen and shark chondroitin-6-sulphate glycosaminoglycan covered by a silicone membrane [9,29]. Permacol is made of porcine dermis, Matriderm^®^ is made of a matrix of bovine type I collagen with elastin, and Oasis is derived from porcine intestinal submucosa [17,18].

Some systems have added cells. Integra can be in this category, if seeded with keratinocytes under the silicone layer [9]. TransCyte™ is similar to Biobrane^®^ but is seeded with fibroblasts cultured from neonatal human foreskin [24,25]. Dermagraft, also uses neonatal foreskin fibroblasts cells but seeded on a biodegradable polyglycolic acid mesh [4,17,18]. Apligraf^TM^ also uses fibroblasts from neonatal foreskin seeded into bovine collagen, which is exposed to heat to produce a loose fibrous network [4,17,18]. OrCel^TM^ is fibroblasts also seeded into a bovine collagen type I matrix but with keratinocytes cultured on top [4,17,18].

Hyalograft 3D is autologous cultured fibroblasts seeded onto a 3D hyaluronic acid derived scaffold [17,18]. Hyalomatrix^®^ is also autologous cultured fibroblasts seeded on a hyaluronan base scaffold and an outer silicone membrane [17,18]. Laserskin (or Vivoderm) is autologous keratinocytes seeded on an esterified hyaluronic acid matrix [17,18,24]. The TissueTech autograft system, combines Hyalograft 3D and an epidermal substitute (Laser skin) [17,18]. Permaderm™ contains both epidermal and dermal components composed of autologous fibroblasts and keratinocytes cultured on a collagen substrate [17,18].

Cultured epidermal autografts (CEAs) are made of autologous keratinocytes sheets attached to a petrolatum gauze support (Epicel™), sprayed into the wound (Cell Spray or Epidex) or just small keratinocyte sheets cultured from the patient’s follicles (Epidex) [17,18,24,25]. There are also some allogenic living epidermal substitutes such as Stratagraft, Tiscover™. DenovoDerm™ and DenovoSkin™ that are not approved yet in the US [17,18,24,25]. These do not however, fit into BER, serving more as natural grafts [17,18].

#### 3.1.2. Limitations of Commercially Available Skin Substitutes

The main limitations of these commercially available skin substitutes is reduced vascularization, poor mechanical integrity, failure to integrate, scarring, and immune rejection [4,17,18]. Time is also a concern with many techniques requiring two separate applications and/or 2–3 weeks of cell culture before they are ready for use [4,7,17,18]. 

The time for making the devices as well as the storage and sterility issues have made these devices expensive [4,7]. In addition, the development time due in part to long and costly regulatory hurdles add to the cost [7]. For example, in 2016 the cost to cover 1% body surface area with Epicel™ is at more than $13,000 [18,124,125]. 

#### 3.1.3. Future Directions

Due to these limitations work is still ongoing to make more effective and efficient systems. Many of these approaches have been previously covered.

##### Scaffolds

Work is ongoing to further optimize previously mentioned scaffolds as well as other degradable natural and synthetic scaffolds. The approach has been to (1) use natural materials and remove unwanted bioactivity or (2) use synthetic systems and add bioactivity to make them more biomimetic [17,18,24,25]. Examples include removing the scarring tendency of fibrin scaffolds or adding biochemicals to the surface of synthetic materials (e.g., fibronectin binding sites) [4].

##### Stem Cells

Work will continue with stem cells and progenitor cells [4]. As a result of legal, religious, cultural, regulatory, and ethical concerns, research strategies have focused on autologous stem cell therapies [4,17,18]. Although not as flexible as embryonic stem cells, autologous bone-marrow or blood derived stem cells and progenitor cells are flexible enough to generate a complete skin-like substitute [4,19]. There has also been work on dedifferentiating stem cells like ones found in hair follicles [4]. In any case the difficulty with stem cells is to have them differentiate along the desired path to produce the desired cell line [4]. This is more difficult as the stem cells become closer to the pluripotential embryonic stem cells [4,7]. These cues can be cell–cell, cell-soluble factor, or cell–matrix interactions [7]. There can also be environmental cues (mechanical, electrical, etc.) [4,7]. The biomaterial can supply the cell–matrix interactions (e.g., the fibronectin binding sites previously mentioned) or just be the delivery mechanism of the cells [4,7,19].

Stem cells are being used to grow skin *in vitro*, but also to speed healing in wounds (previously described) [4,19]. As previously discussed the angiogenic response is the rate limiting one for most skin wounds and therefore endothelial progenitor cells (EPCs) have been used [4,7]. Some have found that vasculogenesis can be done with seeding of EPCs with mesenchymal stem cells or even fibroblasts [4,19]. A number of investigators have found that mesenchymal stem cells can help with regeneration of skin structures such as rete ridges, normal collagen architecture in the dermis, and skin appendages including hair follicles [19].

##### Additive Manufacturing

Additive manufacturing (3D printing) is being used to create skin substitutes and wound scaffolds that can be made to fit the contour of the wound plus can control the 3D architecture of cells and the biomaterial [19]. In addition, some are working on building in vasculature either with endothelial lined tubes or, as previously mentioned, seeding EPCs and stem cells to induce vasculogenesis [19]. Biomolecules can also be part of the 3D printing process [4,19].

##### Skin Substitutes versus Scaffolds

There is a continuum between scaffolds and skin substitutes, which can be more easily controlled with 3D printing and the use of stem cells [4,7]. Skin substitutes are to mimic skin grafts, which again get remodeled and act like scaffolds to a degree [4]. Scaffolds serve as a framework to help regenerate the native skin structure *in vivo* [4,7]. A skin substitute tries to create the native skin structure either *in vitro* or via 3D printing [4,7]. So the continuum is how much of the native structure is present prior to implantation [4,7]. There is also how much of the native structure is available when it is developed *in vitro* [4,7]. In a sense it mimics the continuum between scaffolds and skin substitutes *in vivo* [4,7]. So, the combo of stem cells and 3D printing allow control of how much structure needs to be grown *in vitro* as well as *in vivo* [4,7].

##### Whole-Organ Decellularization

Although decellularized tissue has been used for a while we are getting better at whole organ decellularization, providing a more biomimetic structure than other synthetic scaffolds [19]. The current process uses a detergent perfused through the native blood vessels, to solubilize and remove cellular components (i.e., intracellular proteins and nucleic acid material), which generates an acellular whole-organ scaffold with normal blood vessel architecture (minus the endothelium) [19]. Preliminary studies have been done with kidneys, hearts, and lungs [19].

Once decellularized it becomes a biomaterial and the question becomes where on the continuum of scaffold versus graft it should fall [4]. Repopulating cells, especially blood vessel endothelium has been a challenge as well as how much of this process occurs *in vitro* [4].

##### Overall Strategies

Although I have outlined general strategies as well as specific strategies I have worked on, there is a need to prioritize strategies. This is to be cost effective in our spending on research to both go after strategies that are most promising as well as most likely to be commercialized [7]. There is also a need to tailor the design to different pathophysiologies [4]. In many cases, if the insult is short term or can be removed (as well as any compromised tissue) skin wound strategies can be similar. Some disease states (e.g., diabetes) however can’t be cured presently (although controlled to a certain extent) and wound repair and regeneration can be compromised by reduction in angiogenesis and/or supply of stem cells [4,7]. The pathophysiology can therefore alter the device design constraints as well as the need for added biologics and thus alter the regulatory hurdles [4,7]. 

Since the goal of producing a replacement for an autograft has not been met yet; it appears we should, at this time, concentrate more on scaffolding than skin substitutes (which end up being scaffolds anyway) [4,7]. For reduction in costs (development costs and ultimate costs to the consumer) the procedure should be simple enough to be done at the patient’s home (home-health) or outpatient and have an easy regulatory process [4,7]. This means to minimize the use of non-autologous biologics and the amount of manipulation that occurs *in vitro* [4,7]. In essence reduce the amount of *in vitro* culturing to make a skin substitute and allow the “culturing” to occur *in vivo* as part of the scaffold system [7].

The amount of non-autologous biologics necessary is dependent on the pathophysiology and state of current technology [7]. It ultimately, as previously mentioned, comes down to design constraints for the specific clinical condition and the ability of various techniques to meet these design constraints. Because the techniques are evolving it is still hard to determine the upper limit of each technology [7]. We know some things like the upper limits on some cell activities like replication, migration, protein production, growth factor release, but not all the critical bioprocesses [7,33]. 

In many cases, we start with a more complex system that meets the design constraints and then look at ways to reduce the complexity (to reduce development cost and regulatory hurdles) while still meeting the design constraints [4,7]. Different applications, of course, would have different design constraints for levels of regeneration of structure and function. Once the minimum design constraints are met with the least complex system the question can be asked “is it worth the additional development time, commercialization cost, and ultimate product cost for a better duplication of skin function and or structure? [4,7]”.

For example, in burns, depending on the location there would be different minimal design constraints for skin pliability and aesthetics [4,7]. We are also not good at regenerating skin appendages such as hair follicles or sweat glands [4,7]. Again, the importance of these are different for different applications as well as to help determine whether a skin scaffold or skin substitute can meet these design constraints at all (or at least as good as a skin graft) [4,7].

### 3.2. Current Biomaterial Enhanced Regeneration Techniques for Load Bearing Applications

Again, the emphasis is on degradable/regenerative systems in load bearing applications with fixation of a simple long-bone fracture the specific application targeted. Since the use of degradable/regenerative systems in load bearing applications is limited, some non-biomaterial enhanced regeneration strategies will also be presented. Most BER systems for bone are graft substitutes, which are rarely used in long-bone fractures. For low-load applications there are degradable plates, screws, and pins; which are currently not suitable for long-bones. There are a number of techniques to help in the healing process, which are only BER when used with a biomaterial. Electrical stimulation is one example and a study using it is included in this issue.

#### 3.2.1. Stability

Current clinical treatment is not BER and typically requires metal devices (plates, pins, screws, anchors, wire, etc.) [34]. The main problems with these devices are (1) the high complication rates (15% for internal fixation devices) and (2) that the designs interfere with healing, lengthening the rehabilitation time; particularly if the hardware is to be removed [34,126,127,128,129,130,131]. Many of the complications (e.g., refracture of the bone) can be reduced by speeding healing. In clinical practice, implants are removed (80% of the time in many cases) to speed healing and reduce long-term complications [34,122,128]. This typically requires a second rehabilitation cycle and in many cases leaves holes in the bone, which increases the susceptibility to refracture [34]. There are some polymers and degradable products, but for low- load applications only [16,19,20,21].

For simple transverse fractures of the central portion of a long-bone (diaphysis), there would not be a biomaterial used in between the two fracture surfaces. As the fracture becomes more complicated, however, and there is more space between the fracture surfaces, the use of graft substitutes becomes more likely [19,20,21]. These systems, however, currently are not strong enough to provide much stability by themselves in the early stages of healing [7,34,19,20,21].

#### 3.2.2. Healing

Again, for simple fractures, little is currently done from a biomaterial point of view besides the stability. Additional external factors similar to those covered in the skin section are used such as growth factors, cells, and electrical stimulation [8,19,20,21]. 

Severe fractures with displacement and/or comminution, non-unions, osteonecrosis, or bone cancer can lead to areas of missing bone. This is similar to skin in that the choice is grafting or synthetic bone scaffolds or bone substitutes. There are many examples of scaffolds and bone substitutes used [8,14,15,16,17,19,20,21,126,127], but the emphasis in this review has been on long-bone fractures that do not have defects large enough that they need to be filled-in. 

As previously mentioned, growth factors such as bone morphogenic protein (BMP) as well as mesenchymal stem cells have been added to enhance bone regeneration [19,20,21]. As well as exogenous factors such as electrical stimulation and mechanical loading [8].

#### 3.2.3. Limitations of Commercially Available Load Bearing Systems

##### Mechanical

Again, the issue is the high complication rates for internal fixation devices requiring removal in many cases as well as interfering with healing [34]. Removal typically requires a second rehabilitation cycle and in many cases leaves holes in the bone, which increases the susceptibility to refracture [34]. Current degradable fixation systems do not maintain strength long enough for most orthopedic applications [34,123].

Current bone scaffold systems also do not have sufficient mechanical properties for functional loading and typically require additional fixation [34]. This is further complicated by the importance of mechanical loading for healing [34]. Ultimately, the system (bone plus device/scaffold) should have sufficient mechanical properties throughout the healing process (osteotransduction) [7,8,34]. In most cases we do not have degradable scaffolds or fixation devices that can handle the load initially so we have to rely on permanent devices or work on low or non-loaded cases [34]. 

Part of the mechanical aspect is integration to surrounding bone. We are limited in materials that can set up *in vivo* to fill a defect [19,20,21]. The system either has to be pre-formed or the space has to be closed while the material sets up. In both cases, external fixation or limits on loading need to be instituted until tissue grows into the scaffold [7,34].

##### Healing

For mechanical integration or filling a defect in bone, typically the more loading the faster the healing [7]. However, if the loading is too high the healing area can re-break or separate from the surrounding bone [7]. Also, the better integration between the device and the surrounding bone the better the transfer of loading will be [7]. To prevent “rebreaking” we typically overdesign the fixation systems (since they are currently permanent) meaning they are too stiff to allow ideal loading to the bone healing area; slowing the healing [7,34]. Even if we could design degradable implants that have sufficient mechanical properties during healing, we probably will initially over design them as well until we know the system can handle the loads and then increase the degradation rate slowly in future designs [34]. Similar to skin, as we speed the healing with cells and growth factors the development cost and time increase as well as the final clinical treatment cost.

#### 3.2.4. Future Directions

Similar to skin, work is continuing in providing better scaffolds and graft substitutes. To be used in long-bones they need to be stronger, able to attach and integrate with surrounding tissue, and be able to be formed in situ during surgery [4,7]. For the case of transverse fractures in long-bones, a degradable plate or nail that lasts through most of the healing phase needs to be developed [7,34]. Degradable metals show the most promise currently, but have to be modified to maintain mechanical strength long enough [34,122,123]. 

Similar to skin, I have outlined general strategies as well as specific strategies I have worked on, there is still a need to prioritize strategies. There is also a need to tailor the design to different pathophysiologies. Again, the pathophysiology can therefore alter the device design constraints as well as the need for added biologics and thus alter the regulatory hurdles. 

Also similar to skin, the goal of producing a replacement for an autograft has not been met yet, emphasis should be on scaffolding systems as well as degradable/regenerative systems that can provide the needed stability while allowing loading on the healing bone. Although it will be difficult to get to a point where procedures are simple enough to be done at the patient’s home, the use of non-autologous biologics should be minimized until the development costs and regulatory hurdles allow the benefit gained to be worth the extra time and cost.

Like for skin, because the techniques are evolving it is still hard to determine the upper limit of each technology. Again, there is also the trade-offs between commercializability, time to healing, and level of regeneration; with different applications having different design constraints for levels of regeneration of structure and function. There have been a number of promising clinical trials, but the cost and regulatory hurdles to get these products to market are significant barriers [19,20,21,132,133,134].

## Figures and Tables

**Table 1:** Biocompatibility Design Hierarchy.

Host	Implant
Regeneration	Degradable
Integration	Bioactive
Minimal Inflammation	Inert
Inert	-
